# Prefrontal Lobe Brain Reserve Capacity with Resistance to Higher Global Amyloid Load and White Matter Hyperintensity Burden in Mild Stage Alzheimer’s Disease

**DOI:** 10.1371/journal.pone.0149056

**Published:** 2016-02-12

**Authors:** Ya-Ting Chang, Chi-Wei Huang, Nai-Ching Chen, Kun-Ju Lin, Shu-Hua Huang, Yen-Hsiang Chang, Shih-Wei Hsu, Wen-Neng Chang, Chun-Chung Lui, Che-Wei Hsu, Chiung-Chih Chang

**Affiliations:** 1 Cognition and Aging Center, Departments of Neurology, Kaohsiung Chang Gung Memorial Hospital, Chang Gung University College of Medicine, Kaohsiung, Taiwan; 2 Nuclear Medicine, Kaohsiung Chang Gung Memorial Hospital, Chang Gung University College of Medicine, Kaohsiung, Taiwan; 3 Radiology, Kaohsiung Chang Gung Memorial Hospital, Chang Gung University College of Medicine, Kaohsiung, Taiwan; 4 Department of Nuclear Medicine and Center for Advanced Molecular Imaging and Translation, Chang Gung Memorial Hospital, Taoyuan, Taiwan; Biomedical Research Foundation, UNITED STATES

## Abstract

**Background:**

Amyloid deposition and white matter lesions (WMLs) in Alzheimer's disease (AD) are both considered clinically significant while a larger brain volume is thought to provide greater brain reserve (BR) against these pathological effects. This study identified the topography showing BR in patients with mild AD and explored the clinical balances among BR, amyloid, and WMLs burden.

**Methods:**

Thirty patients with AD were enrolled, and AV-45 positron emission tomography was conducted to measure the regional standardized uptake value ratio (SUVr) in 8 cortical volumes-of- interests (VOIs). The quantitative WMLs burden was measured from magnetic resonance imaging while the normalized VOIs volumes represented BR in this study. The cognitive test represented major clinical correlates.

**Results:**

Significant correlations between the prefrontal volume and global (r = 0.470, p = 0.024), but not regional (r = 0.264, p = 0.223) AV-45 SUVr were found. AD patients having larger regional volume in the superior- (r = 0.572, p = 0.004), superior medial- (r = 0.443, p = 0.034), and middle-prefrontal (r = 0.448, p = 0.032) regions had higher global AV-45 SUVr. For global WML loads, the prefrontal (r = -0.458, p = 0.019) and hippocampal volume (r = -0.469, p = 0.016) showed significant correlations while the prefrontal (r = -0.417, p = 0.043) or hippocampal volume (r = -0.422, p = 0.04) also predicted better composite memory scores. There were no interactions between amyloid SUVr and WML loads on the prefrontal volume.

**Conclusions:**

BR of the prefrontal region might modulate the adverse global pathological burden caused by amyloid deposition. While prefrontal volume positively associated with hippocampal volume, WMLs had an adverse impact on the hippocampal volume that predicts memory performance in mild stage AD.

## Introduction

Although amyloid deposition in Alzheimer's disease (AD) is widely accepted to represent a central pathological mechanism [1], recent meta-analysis and reviews suggested that a certain amount of normal cognitive elders also harbor intracerebral beta-amyloid deposits [2–4]. One possible explanation is that although amyloid positivity may be necessary in AD diagnosis, the rate of cognitive decline is driven by the neurodegenerative process. The notion has been validated recently by serial amyloid and magnetic resonance imaging studies [5]. Another possible explanation may be related to the compensatory mechanisms that serve as a protective buffer. In the literature, both brain reserve (BR) and cognitive reserve have been mentioned [6, 7]. The cognitive reserve emphasizes the premorbid functional reserve, while the BR implies differences in the quantity of available neural substrate [8, 9] that reduces the pathological impacts [10].

A higher prevalence of dementia patients having a smaller total brain volume supports the BR hypothesis [9] while the structural determinants with related genetic effects were established in a twins study [11]. Using direct fibrillary amyloid β pathology measurement or amyloid positron emission tomography (PET) quantification, neuroimaging evidence for the BR against amyloid burden was reported [12, 13]. In two groups of study populations showing equivalent pathological burden, the major determinants for cognitive integrities were found to be related to the volume of hippocampus or total intracranial volume (TIV) [12]. Another study suggested that a larger temporal lobe volume provides BR that shows resistance to fibrillar β-amyloid impact within the gray matter (GM) [13].

In addition to neurodegenerative cascades, the coexisting cardiovascular risk factors in patients with AD represent another pathological burden that carries clinical impacts. The small vessel disease in AD can be visualized as hyperintense white matter lesions (WMLs) on T2-fluid attenuated inversion recovery magnetic resonance images (MRI) [14]. WML burden has been shown to greatly modulate the pathological progression and cognitive decline in AD [15, 16]. As WML and amyloid burden both indicated intracerebral pathological impacts, the elucidation of regional BR that protects against each pathological burden may help to understand their clinical weightings.

The issue of appropriate head size adjustment has been reported in the context of cortical or WML structure changes in the elderly [17]. Because of methodological concerns, normalization of regional brain volume by TIV (i.e., TIV-adjusted volume of interest [VOI]) is often performed in studies on degenerative disease degenerative studies [18]. The WML severity can be quantitatively measured by the hyperintense signal volume in MRI or by a visually rated scale [19]. The volumetric quantification of WML burden provides an unbiased measurement of lesional load. However, as this often requires computational analysis, visual rating assessment is still more commonly used in clinical trials [19].

In an autopsy-verified histopathological study, fibrillar Aβ load in AD was assessed with high sensitivity and specificity using florbetapir (AV-45)-labeled positron emission tomography (PET) imaging [20]. The present study aimed to explore the clinical balance between protective and pathological mechanisms in patients with mild AD. The protective roles of regional BR against two pathological mechanisms (i.e., amyloid and WML loads) were included in the analysis. A role for regional volume showing BR would predict that, when controlling for disease severity, AD patients with larger regional volume would have more advanced pathological changes such as amyloid load or WML burden.

## Methods

### Inclusion and Exclusion Criteria

Thirty patients with AD were enrolled from the Cognition and Aging Center at the Department of Neurology of Chang Gung Memorial Hospital from 2011 to 2014. Subjects were included on the basis of consensus of panels composed of neurologists, neuropsychologists, neuroradiologists, and experts in nuclear medicine [21, 22]. AD was diagnosed according to the International Working Group criteria [23] with a clinical diagnosis of typical AD. All of the AD patients were under stable treatment with acetylcholine esterase inhibitors from the time of diagnosis. A Clinical Dementia Rating (CDR) score of 0.5 or 1 represented mild-stage AD in this study. The exclusion criteria were a history of clinical stroke, a modified Hachinski ischemic score >4 [24], and depression.

### Study Design

The study was approved by Chang Gung Memorial Hospital's Institutional Review Committee on Human Research, and all of the participants and their authorized caregivers provided written informed consent. AV-45 PET scans, cognitive testing, and MRI were all performed within a duration of 4 weeks.

### MRI Acquisition and Cortical Volumetric Analysis

MRI images were acquired on a GE 3T Signa Excite scanner (GE Medical System, Milwaukee, WI). The scanning protocol included: 1) fluid attenuated inversion recovery, turbo spin-echo sequence with repetition time/echo time/flip angle: 9000 ms/85 ms/180°, 240 × 240 mm field of view, 320 × 224 matrix, and 34 slices with a thickness of 4 mm were acquired in 2 minutes and 44 seconds.; and 2) T1-weighted, inversion-recovery-prepared, three-dimensional, spoiled, gradient-recalled acquisition in a steady-state sequence with repetition time/inversion time of 8,600 ms/450 ms, 240 × 240 mm field of view, and 1-mm slice thickness.

**Fig 1:**
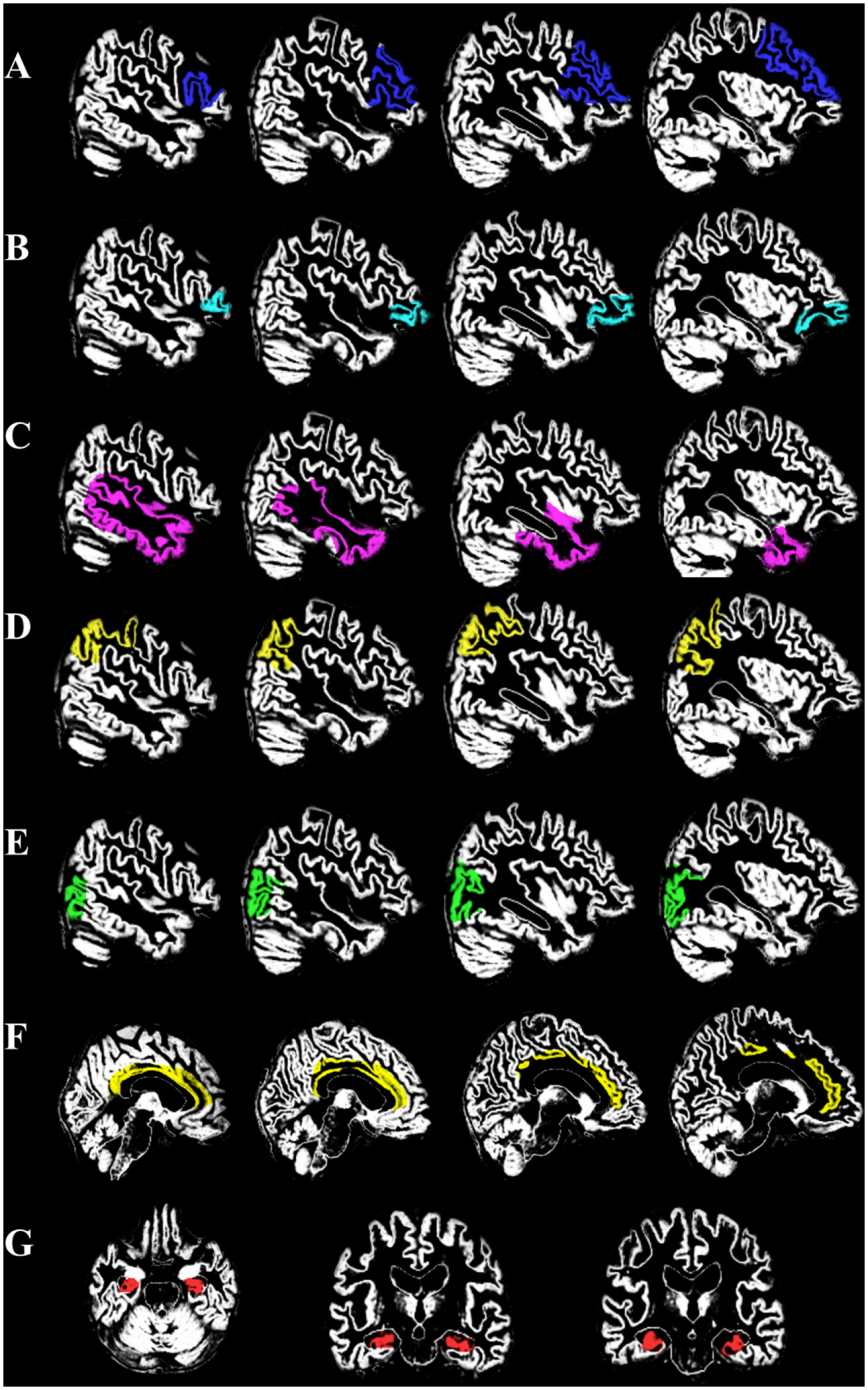
Illustration of volume of interest (VOI) overlying on segmented gray matter. (A) prefrontal lobe (B) orbitofrontal (C) lateral temporal (D) posterior parietal (E) occipital (F) cingular cortex (G) hippocampus.

Using the Statistic Parametric Mapping software version 8 (http://www.fil.ion.ucl.ac.uk/spm/software/), the preprocessing of T1 MRI involved removal of non-relevant tissue, intensity and spatial normalization to the MNI space, and tissue segmentation. We used the Individual brain Atlases using Statistical Parametric Mapping (IBASPM) (http://www.thomaskoenig.ch/Lester/ibaspm.htm) [25, 26] for regional labeling and volumetric calculations. The regional labeling was identified after aligning to the 116 automatic anatomical label (AAL) structures and the volume of each identified structure was calculated using the IBASPM toolbox of *Volume Statistic*. Eight cortical VOIs were defined (Fig 1), including the lateral prefrontal (i.e., superior, superior medial, middle, inferior opercula, inferior triangular frontal regions), orbitofrontal, anterior cingulate cortex, parietal, lateral temporal (i.e., superior, middle, and inferior lateral temporal regions), hippocampus, and occipital cortical regions. Using *Segmentation* in IBASPM, the images were segmented into cerebrospinal fluid (CSF), GM, and white matter (WM). The raw VOI volume and TIV were calculated based on the *Volume Statics* result from IBASPM. TIV represented the sum of GM, WM, and CSF volumes, and the VOI statistics were made controlled for TIV [27].

### WML Assessment

**Fig 2:**
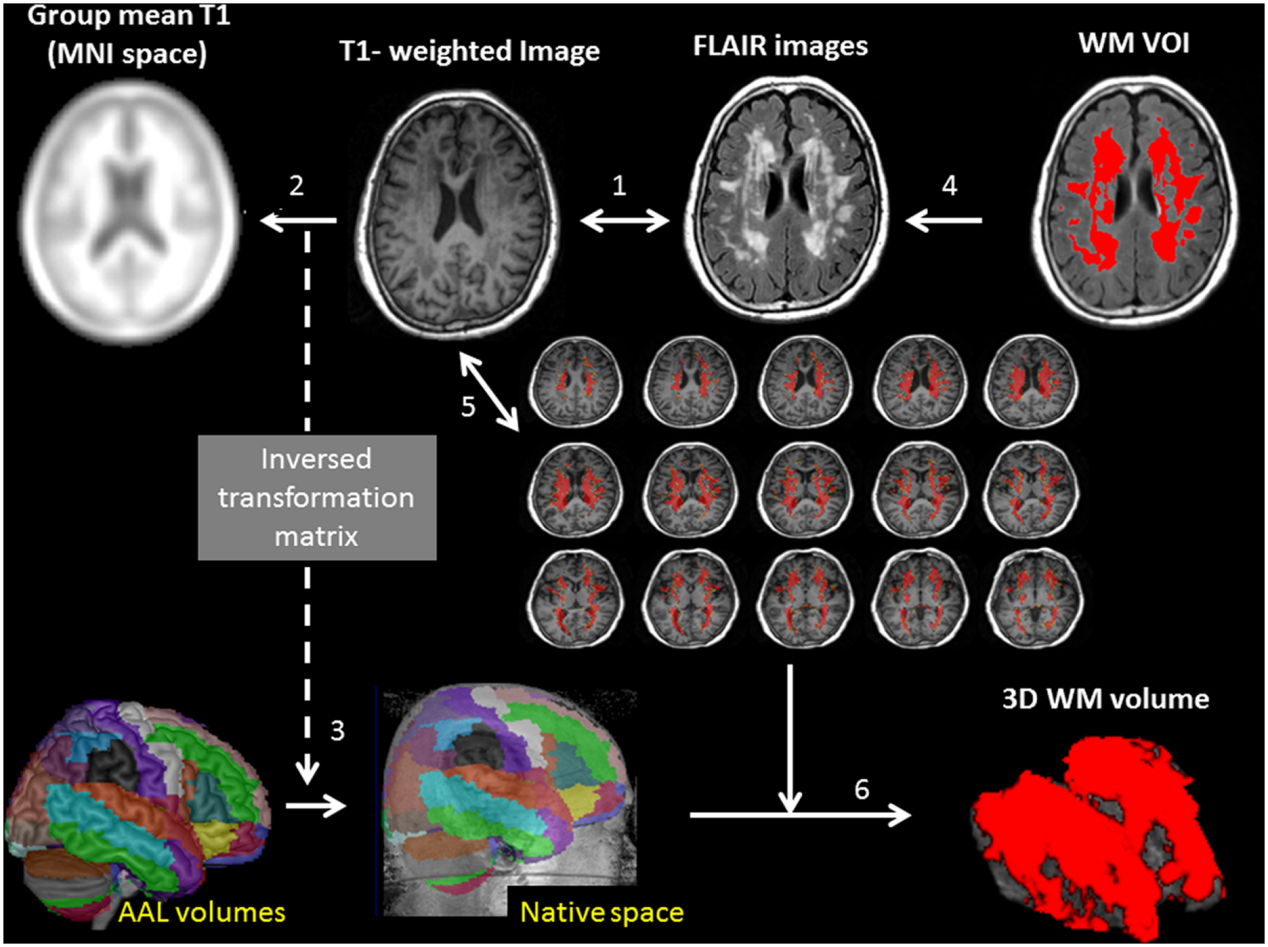
Illustration of automatic quantification of 3D white matter (WM) lesion burden. (1) Individual T1-weighted image were registered to the corresponding FLAIR images using a 12 degrees of freedom affine transformation. (2) To obtain the transformation matrix, the coregistered T1-weighted images were registered to the averaged customized group T1 template in MNI space. (3) The inverse transformation matrix from step 2 was applied to the AAL template to generate corresponding AAL volumes in each individual's 3D T1WI native space for later calculation of normalized 3D WM volume. (4) WM volume of interest (VOI) on FLAIR sequences (5) Transfer the white matter volume of interest to the corresponding T1 image. (6) 3D WM volume constructions.

For the quantification of WML burden, we calculated the total WML volume using self-developed pipeline and the lesion Segmentation Toolbox image processing software [28], which was developed for use under the Statistical Parametric Mapping and achieved good agreement with manual tracing with R^2^ values of 0.93. Using the fluid attenuated inversion recovery sequence, the software automatically classified areas of abnormal WML from normal white matter (Fig 2). The total volume of the high intensity WML in each subject was extracted, and WML burden was further analyzed after controlling for TIV.

In addition to the automatic method, we also used the visually-rated Age-Related White Matter Changes (ARWMC) rating scale for cross-validation [19]. As the ARWMC scale represented ordinal measures rather than a continuous scale and to increase the statistical power, we used the WML category cutoff values proposed by de Leeuw *et al*., [29] with a mild WML defined as an ARWMC of 1 to 4, and a moderate to severe WML as an ARWMC score of ≥ 5 [29]. These WML categories were chosen because a previous study suggested that these cutoff values could accurately predict hippocampal atrophy [29].

### AV-45 PET Acquisition and Analysis

AV-45 was synthesized at the cyclotron facility of Chang Gung Memorial Hospital. The PET acquisition protocol, optimal scanning time, and image reconstruction followed a previous report [30]. A single intravenous bolus of approximately 370 MBq (10 mCi) of [18F] AV-45 was injected, followed by a saline flush. In brief, helical computed tomography images were obtained for attenuation correction at 40 minutes. Each PET acquisition consisted of two 5-minute dynamic frames obtained 50 minutes post-injection in 3D mode using a Biography mCT PET/computed tomography system (Siemens Medical Solutions, Malvern, PA, USA). Summed images were subsequently created for further analysis.

The PET images were first co-registered to the 3-dimensional T1 images by linear transformation using Statistical Parametric Mapping 8 software. The global GM AV-45 load was defined from the MRI GM-segmented images, and the cerebellar GM represented the reference region. The standardized uptake value (SUV) was related to the injection dose and normalized to body weight. The SUV ratio (SUVr) was calculated by determining the ratios of SUV between the global GM and the reference cerebellar cortical region.

### Neuropsychological Assessments

A trained neuropsychologist administered the following tests: Mini-Mental Status Examination (MMSE), Clinical Dementia Rating scale Sum of Boxes (CDR-SB) score, and the 11-item Chinese version of the Alzheimer’s Disease Assessment Scale-cognitive subscale (ADAS-cog) [31]. As episodic memory represents the salient feature of AD, composite memory score was assessed by adding the memory scores across the CDR subcategory of memory and ADAS-cog memory test battery, and each measure in the battery carried the same weight.

### Statistical Analysis

All values were expressed as mean±standard deviation (SD). To assess the relationships among continuous variables including GM SUVr, regional volume, and WML volume, Pearson's rank correlation coefficients were calculated with a corresponding two-sided significance test at the 0.05 significance level. To assess the appropriateness of using parametric statistics for these analyses, we used the Kolmogorov-Smirnov test to examine the normality, and *p* values > 0.05 indicated no significant deviation from normality. All of the cognitive and imaging variables were normally distributed. The MMSE, ADAS-cog, CDR sum of box (CDR-SB), and composite memory scores were used as indices for controlling the cognitive severity in the statistical models. Stepwise regression analysis was carried out to determine the best predictors of total GM AV-45 SUVr. The strategy for the regression analysis was to assess the association between GM amyloid load and regional cortical volume. Each model used cognitive evaluation test scores, TIV, age, education, gender, and cortical volume as independent variables. For the relationship between regional BR and WML, we used partial correlation and stepwise regression analyses. The cognitive reserve of the AD patients was measured by cognitive outcomes as dependent variables and the educational level, gender, age, and TIV as independent variables. All of the statistical analyses were conducted using the Statistical Package for Social Sciences software package (version 18 for Windows^®^, SPSS Inc., Chicago, IL), and a p value less than 0.05 (two-tailed) was considered to be statistically significant.

## Results

### Demographic and Clinical Characteristics

Thirty patients completed the study. Their demographic, clinical, and neuroimaging variables are presented in Table 1. In patients with CDR 0.5, 17 patients had a mean age of 74.4±7.5 years, education of 9.1±4.9 years, MMSE of 20.4±3.7, and ADAS-cog of 35.0±14.0, and in patients with CDR 1, 13 patients had a mean age of 79.5±8.1 years, education of 8.0±6.2 years, MMSE of 16.5±3.6, and ADAS-cog of 42.8±13.3.

**Table 1:** General characteristics of the Alzheimer's disease patients.

Clinical and demographic characteristics	Mean (standard deviation)
CDR-SB	0.72±0.36
Composite memory score	8.2±1.7
TIV-adjusted cortical volume	
Prefrontal	0.05±0.01
Orbitofrontal	0.02±0.01
Lateral temporal	0.07±0.01
Parietal	0.05±0.01
Occipital	0.05±0.01
Hippocampal	0.004±0.001
Anterior cingulate cortex	0.007±0.001
Posterior cingulate cortex	0.002±0.0004
Raw cortical volume (cc)	
Prefrontal	66.0±14.6
Orbitofrontal	27.9±8.1
Lateral temporal	86.6±13.5
Parietal	60.2±9.0
Occipital	63.3±10.1
Hippocampal	4.9±1.3
Anterior cingulate cortex	8.9±1.4
Posterior cingulate cortex	2.0±0.5
Pathological marker 1	
GM AV-45 values (SUVr)	1.3±0.3
Pathological marker 2	
ARWMC scores	5.0±3.5
Normalized WML loads	0.02±0.01

Parametric continuous variables presented as mean (standard deviation). ADAS-cog, Alzheimer's Disease Assessment Scale-Cognitive scale; ARWMC, Age-Related White Matter Changes Rating Scale; CDR-SB, clinical dementia rating sum of boxes; GM, grey matter; PET, positron emission tomography; SUVr, standardized uptake value ratio; TIV, total intracranial volume; WML, white matter lesions.

### Volume in the Prefrontal Lobe Independently Determined AV-45 SUVr

Based on the aforementioned finding, we further analyzed the effects of 8 VOIs volumes using partial correlation and multiple regression models controlling for age, gender, education, TIV, and cognitive test scores of the MMSE (Table 2). The lateral prefrontal volume repeatedly showed a positive correlation with global GM AV-45 SUVr after controlling for age, gender, education level, TIV, and MMSE (partial *r* = 0.558, *p* = 0.024). Further correcting for multiple comparisons, we ran a multiple linear regression model that had all of the regions in it. A positive correlation was observed between prefrontal volume and global GM SUVr (β = 0.558, P = 0.02) after adjusting for age, gender, TIV, and MMSE.

**Table 2:** Partial correlations between cortical volume and GM AV-45 SUVr in 30 patients.

	Model 1 ^a^	Model 2 ^b^
Regional cortical volume	Global GM AV-45 SUVr	Global GM AV-45 SUVr
**Prefrontal**	0.470*	0.588*
**Orbitofrontal**	0.181	0.210
**Posterior parietal**	0.071	0.079
**Lateral temporal**	0.308	0.331
**Occipital**	0.079	0.082
**Hippocampal**	0.224	0.242
**Anterior cingulate cortex**	0.045	0.050
**Posterior cingulate cortex**	0.250	0.268

^a^Partial correlations controlled for age, gender, education, TIV and Mini-Mental Status Examination (MMSE). Data present as partial correlation r.

^b^Multiple regression analysis controlled for age, gender, education, TIV and MMSE. Data present as β coefficient.

* p<0.05

AD, Alzheimer's disease; GM, gray matter; SUVr, standardized uptake value ratio; TIV, total intracranial volume;

The regional BR of prefrontal lobes with regards to global GM amyloid was rechecked using the regression model controlling for age and one of the 4 cognitive tests (S1 Table). The results showed a significant independent role of the prefrontal lobe in global GM AV-45 load (*p*<0.05).

### Impact of WML Load on Cortical Volume

We then investigated the influence of WML load on the eight cortical VOIs volume. Controlling for age, gender, and TIV, only the hippocampal volume (r = -0.469, p = 0.016) and prefrontal volume (r = -0.458, p = 0.019) correlated significantly with global WML volume. In a multiple regression model, WML load independently associated with hippocampus volume (β = -0.434, P = 0.016).

In the predefined ARWMC groups [29], a significantly larger TIV-adjusted prefrontal volume was observed in the mild WML group (n = 13, 0.046±0.006) compared to the moderate to severe WML group (n = 17, 0.034±0.004, p = 0.021). There were no significant differences in age (p = 0.650), the hippocampal volume (mild group: 0.004±0.001, moderate-to-severe: 0.003±0.001; p = 0.08), or in the other 5 VOIs (all p>0.05). Further, prefrontal volume was positively associated with hippocampal volume after controlling for age, gender, and TIV (r = 0.617, p = 0.001).

### Prefrontal Subregions Showing BR to Amyloid and WML loads

As the prefrontal lobes showed BR to both amyloid and WML burden, the significance of individual prefrontal subregions that determined the results were further explored using partial correlation analysis controlling for age, education, gender, TIV, and MMSE (Table 3). The results suggested that the BR capacity of superior prefrontal (*r* = 0.572, p = 0.004), superior medial prefrontal (*r* = 0.443, p = 0.034), and middle prefrontal (*r* = 0.448, p = 0.032) regions determined the resistance to global GM AV-45 SUVr. In comparison, there were no direct correlations between the aforementioned regional SUVr and local volume measures. Meanwhile, only the middle prefrontal volume showed relationships with WML loads. In multiple regression model, only superior prefrontal volume was independently associated with global GM AV-45 SUVr (β = 0.687, P = 0.004).

**Table 3:** Prefrontal regions showing brain reserve to amyloid burden or white matter lesion loads.

	Regional Cortical Volume				
	Superior prefrontal	Superior medial prefrontal	Middle prefrontal	Inferior Operculum	Inferior triangular frontal
**Pathological marker 1**					
**Superior prefrontal SUVr**	0.189;0.388	0.079;0.718	0.074;0.736	-0.078;0.722	-0.083;0.706
**Superior medial prefrontal SUVr**	0.152;0.489	0.099;0.652	0.066;0.765	-0.187;0.393	-0.184;00401
**Middle prefrontal**	0.166;0.449	0.087;0.694	0.044;0.844	-0.127;0.562	-0.102;0.643
**Inferior Operculum frontal SUVr**	0.352;0.099	0.276;0.203	0.227;0.299	-0.083;0.707	0.012;0.955
**Inferior triangular frontal SUVr**	0.276;0.203	0.231;0.290	0.191;0.382	-0.169;0.441	-0.098;0.656
**Global SUVr**	**0.572;0.004**	**0.443;0.034**	**0.448;0.032**	0.130;0.553	0.195;0.372
**Pathological marker 2**					
**Normalized WML**	-0.335;0.118	-0.310;0.150	-0.401;0.058	-0.018;0.936	-0.041;0.853
**ARWMC scores**	-0.337;0.116	-0.309;0.151	**-0.421;0.046**	-0.162;0.459	-0.077;0.728

Partial correlation analysis controlled for age, education, gender, total intracranial volume and mini-mental state examination scores

ARWMC, Age-Related White Matter Changes Rating Scale; SUVr, standardized uptake value ratio; WML, white matter lesions

Data present as partial correlation *r*; p value;

The BR capacities in the superior prefrontal, superior medial prefrontal, and middle prefrontal regions were also significant after controlling for the other 3 cognitive test scores (S2 Table). The middle prefrontal BR against normalized WML loads, however, was found only after controlling for ADAS-cog score.

### Independent Relation of Two Pathological Markers to Prefrontal Volume

As the global GM AV-45 SUVr and global WML load were both related to the prefrontal lobe volume, we investigated the interactions between these two pathological markers. The results showed that the GM SUVr and WML load were independently related to the prefrontal volume, and that there was no interaction between them (dependent variable = TIV-adjusted prefrontal lobe volume; independent variable 1 [X1] = GM SUVr, β coefficient = 6.079, p = 0.022; independent variable 2 [X2] = WML loads, β coefficient = 7.435, p = 0.012; interaction of X1, X2 with β coefficient = 2.669, p = 0.117).

### Prefrontal Volume Reflected BR and Not Focal Swelling

A larger prefrontal volume may represent BR capacity or tissue edema related to amyloid-induced reactive inflammatory responses [13]. We found that after controlling for age, gender, education level, and TIV, the prefrontal volume correlated with composite memory score (*r* = -0.417, *p* = 0.043) and CDR-SB (*r* = -0.433, *p* = 0.035), suggesting that better cognitive performance scores were associated with a larger prefrontal volume. Meanwhile, the relationships between prefrontal GM AV-45 SUVr and prefrontal volume was insignificant (*r* = 0.312, *p* = 0.121).

### Other Regional Volumes that Predicted Cognitive Outcomes

To investigate the relationships between other regional volumes and cognitive outcomes, we performed a partial correlation analysis after controlling for age, education level, gender, and TIV. While lower composite memory score, CDR-SB, and ADAS-cog indicated better memory and cognitive performance, hippocampal volume correlated inversely with composite memory score (*r* = -0.422, p = 0.040) while the parietal volume correlated inversely with CDR-SB (*r* = -0.435, p = 0.034) and ADAS-cog (*r* = -0.474, p = 0.019). None of the other regional volumes were associated with cognitive performance (p>0.05).

### Cognitive Reserve showed no protection against GM amyloid load

As educational level is widely considered to be an important parameter for cognitive reserve [32], we tested whether the educational level may predict the GM AV-45 SUVr after controlling for age and cognitive test scores of the MMSE, ADAS-cog, CDR-SB, and composite memory score. The results were not significant (p>0.05).

## Discussion

### Major Findings

**Fig 3:**
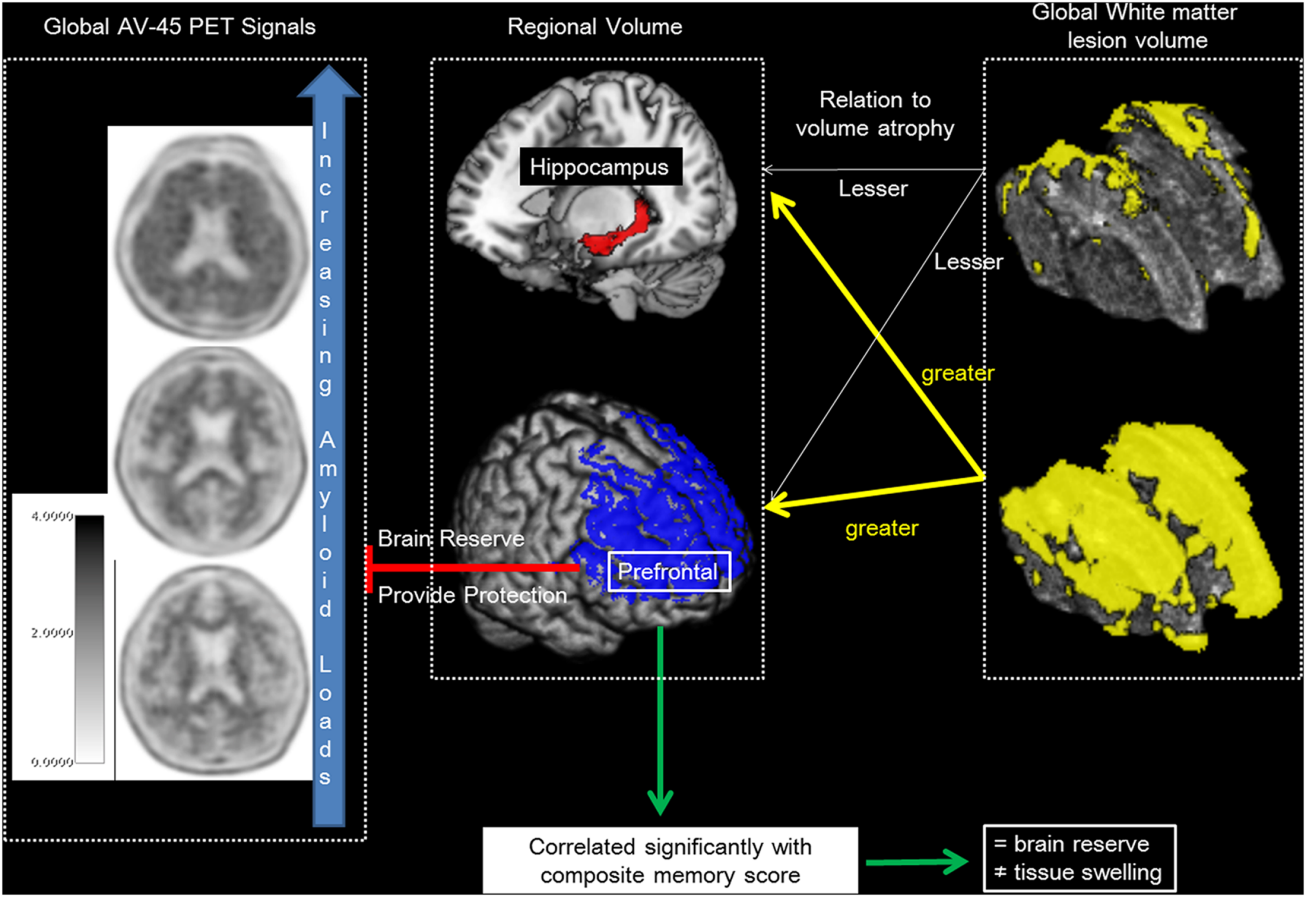
Model of prefrontal brain reserve with related regional volumes, white matter lesions and amyloid pathological load.

This study investigated the clinical weightings among regional BR, amyloid burden, and WMLs in patients with mild stage AD. To facilitate the discussion, we summarized the results model from the statistical analyses (Fig 3). First, a positive association between global AV-45 SUVr and the size of the prefrontal volume, especially the superior, superior medial, and middle prefrontal subregions, was found. As there the relationship between regional volume and AV-45 SUVR was lacking, selective regional prefrontal BR against global amyloid burden was suggested. Meanwhile, the larger prefrontal volume represented BR, and not tissue swelling, since the prefrontal volume significantly predicted the memory score. Second, we found inverse relationships between global WML load and prefrontal and hippocampal volumes. As the prefrontal and hippocampal volumes both were related to memory scores, a greater WML load that linked with smaller prefrontal or hippocampal volume may lead to lower cognitive performance. Of specific note, the adverse impact of WML may be more significant in the middle prefrontal region. Finally, as there were no interactions of the global AV-45 SUVr and WML load with the prefrontal lobe volume, the protective buffer from the prefrontal lobe was independently resistant to these two pathological burdens.

### Mechanistic Delineation of Resistance to Amyloid Pathology Afforded by the Prefrontal Cortex Volume

As amyloid deposition is associated with neurodegeneration [33], the results of the current study and others [12, 13] support the BR hypothesis that may counterbalance the amyloid downstream cascades. As the BR hypothesis emphasizes the intactness of neurons with available synapses [9], a critical time point to observe the existence of regional BR capacity may be prior to the threshold levels of overt dementia. The BR concept also helps to explain why a broad spectrum of cognitive outcomes was observed on the basis of similar amyloid pathology.

In elderly subjects with cognitive deficits, the prefrontal region often serves as the main buffer target [34]. Moreover, as prefrontal lobe activity discriminates prodromal AD patients with rapid conversion versus stable groups, the intactness of prefrontal activity may indicate resistance to disease cascade [35]. Another study reported that hippocampal atrophy may be associated with prefrontal hyperperfusion, suggesting a compensatory mechanism of the prefrontal region in AD [36]. When the early targets such as the hippocampus or temporal regions are disabled, regions such as the prefrontal lobes may offer protection because they are affected by neurodegeneration at a later stage.

In patients with mild cognitive impairment, amyloid deposition in the hippocampus indicates a more likely path towards dementia. An initial increase followed by a subsequent decline in hippocampal activity delineates the biphasic model of hippocampal neuronal compensation responses to amyloid-triggered excitoxicity [37]. Moreover, the temporal lobe was also found to modulate the impact of amyloid plaque on cognition such that normal subjects with larger temporal lobes have lesser susceptibility to Aβ-induced cognitive impairment than those with smaller temporal lobes [13]. Our analysis on the regional volume demonstrated a trend of positive correlation between the temporal lobe and Aβ load in mild AD. Therefore, the present study may provide evidence for the biphasic model of temporal lobe as a BR in mild AD. As the BR-modulation of amyloid-related cognitive decline is initially within the temporal lobe followed by the prefrontal volume in mild AD, regions showing a protective buffer may be relatively dynamic depending on the disease burden. However, longitudinal studies are needed to validate this hypothesis.

In this study, only BR served as the major compensatory region buffering fibrillar amyloid burden, especially in the superior, superior medial, and middle prefrontal regions. While we also investigated cognitive reserve capacity using educational level as the determinant, the effect of cognitive reserve on amyloid load here can be regarded as relatively minor. It is worth pointing out that other factors representing cognitive reserve capacity such as life activities and intelligence quotient were not included in this study. Therefore, although our results did not demonstrate a significant protective effect of educational level, this does not disprove the role of cognitive reserve in mild AD.

### Alternative Hypothesis for a Larger Prefrontal Volume

In transgenic mouse models [38] and human studies of AD [39–41], evidence of glial activation surrounding the amyloid deposition has been found in the cortices. Therefore, a larger regional volume can be related to edema or inflammatory responses triggered by amyloid deposition [13]. As a larger prefrontal volume correlated with better cognitive performance, we considered that a larger prefrontal volume in fact reflected BR and not tissue edema [39]. In addition, typical regions showing glial activation related to amyloid toxicity include the orbitofrontal, temporal, parietal, and occipital associative cortices [39]. While the prefrontal lobes are traditionally not the target regions for highest glial activation, the larger volume in prefrontal lobe found in the current study may argue against the microglial activation and neuroinflammation hypotheses. Meanwhile, the volume of prefrontal lobes showing no correlation with the AV-45 SUVr levels also did not establish the relationships.

### Detrimental Effect of WML on Regional Volume Change

Although neurodegeneration in the GM is the main proposed model of AD, growing evidence highlights the adverse impacts of WMLs [14, 15]. In this study, inverse relationships were found between global WML load and prefrontal volumes, especially the middle prefrontal region. As the prefrontal lobe volume also determines the cognitive outcomes, the global WML loads may disrupt the BR of prefrontal lobe. The presence of WMLs, which attenuates psychomotor speed and complex mental processes [42], may also be mediated by the adverse impact on the prefrontal regions. Our results also suggested an inverse relationship between global WML load and hippocampal volume, which is consistent with previous reports [29, 43–45]. As amyloid deposition starts relatively early in the hippocampus, the relationship between WML load and hippocampal volume in this study suggests an additive pathological burden [46].

### Prefrontal BR, WML, and Amyloid Burden

According to the IWG-2 criteria [47], the proper use of clinical biomarkers can effectively differentiate AD and cerebrovascular diseases. In this regard, we only included AV-45-positive AD patients with typical clinical features. Despite the proper selection, the results still emphasized the clinical significance of mixed vascular pathology. By incorporating two different methods of WML load in this study, we were able to recheck the effect of WML.

In investigating the relationship between prefrontal volume and amyloid deposition, our results suggested that larger prefrontal volume set mild demented patients with higher amyloid pathology load apart from patients with lower amyloid pathology. Based on our study result, larger prefrontal volume with greater amyloid burden indicated the presence of BR in neuroprotection. However, as an inverse relationship between WML load and prefrontal volume was also reported here, higher WML load would lead to greater prefrontal atrophy, possibly related to the disconnection of fiber tracts with the association areas [29]. The impact of WML on the prefrontal volume showed no interaction with the effect of amyloid deposition, and therefore WML load and amyloid burden can be treated as two independent risk determinants. The relation was also independent from age and TIV influence on prefrontal volume.

### Methodological Consideration

This study used GM segmentation to calculate VOIs, which may be more specific in detecting gyral atrophy compared with the use of an anatomical template. It is well known that changes in hippocampal formation are disproportionate to other GM structures in AD [48]. The use of a TIV-adjusted method may mask changes related directly to pathology or in tissues with small volumes. Therefore, we suggest manual rechecking to ensure correct segmentation in regions with small volumes. Meanwhile, the AV-45 signals were extracted by co-registration with the corresponding MRI images in this study. However, correlations between volumetric measurements and quantification of the amyloid burden focusing on the corresponding GM would theoretically be more straightforward. Although a recent report suggested that white matter histogram analysis [49] can also significantly discriminate between patients with AD and healthy subjects, which may improve the standard SUVr method in GM [50, 51], we did not include analysis on white matter based on the rationale that AV-45 binding in the white matter is of a non-specific lipophilic nature.

### Limitations

This study has 3 limitations. First, the sample size is small, which may have led to type I and type II errors. A larger sample dataset validation will be needed to confirm our preliminary results. However, independent relationships of prefrontal lobe, BR, and VOI volume with WML load were found after careful statistical examination by group stratification, correlation analysis, stepwise regression analysis, and rechecking the cognitive score. Second, according to the BR theory, our results proposed that a larger prefrontal lobe would modulate the amyloid plaque impact on cognition in patients with mild AD. However, the interpretation was based on the cross sectional study design and small sample size. Longitudinal data analysis is required to establish the BR capacity in distinct anatomical structures along with the disease course. Lastly, the global AV-45 SUVr was extracted from the total cerebral GM which is likely suboptimal, as some cortical (occipital, medial temporal, sensori-motor) and subcortical (thalamus) regions usually do not harbor amyloid depositions. Therefore, although the results were significant, the GM mask used here may not be sensitive enough to reflect the amyloid burden. The study design could be improved if the amyloid topography showing direct clinical significance is more readily available.

### Conclusion

This study suggested dynamic relationships between BR, WML, and amyloid burden in AD. BR of the prefrontal lobe may resist the global amyloid and WML burden while the hippocampal volume was found to be highly affected by WML burden.

## Supporting Information

S1 Table Independent role of prefrontal volume for total AV-45 SUVr. (DOCX)

S2 Table Independent role of 3 prefrontal regions to regional or global amyloid loads and white matter lesion loads. (DOCX)
