# Supplementary material for: Prefrontal Lobe Brain Reserve Capacity with Resistance to Higher Global Amyloid Load and White Matter Hyperintensity Burden in Mild Stage Alzheimer’s Disease
Source: PLoS One. 2016 Feb 12;11(2):e0149056. doi: 10.1371/journal.pone.0149056 (PMC4752238; doi:10.1371/journal.pone.0149056)
Supplement: S1 Table — (DOCX) [file pone.0149056.s001.docx]

|  | **Model 1** | | **Model 2** | | **Model 3** | | **Model 4** | |
| --- | --- | --- | --- | --- | --- | --- | --- | --- |
| **Dependent variable** | Total GM SUVr | | Total GM SUVr | | Total GM SUVr | | Total GM SUVr | |
| **Independent variable** | Beta value | P value | Beta value | P value | Beta value | P value | Beta value | P value |
| **Age** | 0.153 | 0.51 | 0.044 | 0.85 | 0.150 | 0.51 | 0.115 | 0.61 |
| **Gender** | 0.329 | 0.30 | 0.202 | 0.51 | 0.408 | 0.23 | 0.345 | 0.27 |
| **Education level** | -0.216 | 0.41 | -0.217 | 0.39 | -0.267 | 0.30 | -0.213 | 0.40 |
| **TIV** | -0.211 | 0.44 | -0.120 | 0.66 | -0.312 | 0.28 | -0.215 | 0.42 |
| **Prefrontal volumes** | **0.558** | **0.02** | **0.507** | **0.02** | **0.590** | **0.02** | **0.605** | **0.02** |
| **Cognitive performance** | | | | |  | |  | |
| **MMSE** | -0.230 | 0.31 | - |  | - | - | - |  |
| **ADAS-cog** | - |  | 0.290 | 0.17 | - | - | - |  |
| **CDR-SB** | - |  | - |  | 0.273 | 0.24 | - |  |
| **Composite memory score** | - |  | - |  | - | - | 0.318 | 0.17 |

**Supplementary Table 1. Independent role of prefrontal volume for total AV-45 SUVr**

ADAS-cog, Alzheimer's Disease Assessment Scale-Cognitive Subscale; CDR-SB, clinical dementia rating scale sum of boxes; GM, grey matter; MMSE, mini-mental status examination; TIV, total intracranial volume; Beta value=standardized coefficients.
