# Supplementary material for: Prefrontal Lobe Brain Reserve Capacity with Resistance to Higher Global Amyloid Load and White Matter Hyperintensity Burden in Mild Stage Alzheimer’s Disease
Source: PLoS One. 2016 Feb 12;11(2):e0149056. doi: 10.1371/journal.pone.0149056 (PMC4752238; doi:10.1371/journal.pone.0149056)
Supplement: S2 Table — (DOCX) [file pone.0149056.s002.docx]

**Supplementary Table 2. Independent role of 3 prefrontal regions to regional or global amyloid loads and white matter lesion loads**

|  | **Superior Prefrontal region** | | **Superior Medial Prefrontal region** | | **Middle Prefrontal region** | |
| --- | --- | --- | --- | --- | --- | --- |
| **Dependent variable** | Total GM SUVr | | | | | |
| **Independent variable** | Beta value | P value | Beta value | P value | Beta value | P value |
| **Model 1** | 0.624 | 0.006 | 0.472 | 0.038 | 0.470 | 0.041 |
| **Model 2** | 0.756 | 0.002 | 0.578 | 0.025 | 0.586 | 0.023 |
| **Model 3** | 0.723 | 0.003 | 0.562 | 0.024 | 0.585 | 0.020 |
| **Dependent variable** | Regional gray matter (GM) SUVr | | Regional GM SUVr | | Regional GM SUVr | |
| **Independent variable** | Beta value | P value | Beta value | P value | Beta value | P value |
| **Model 1** | 0.251 | 0.285 | 0.136 | 0.574 | 0.143 | 0.541 |
| **Model 2** | 0.306 | 0.233 | 0.157 | 0.561 | 0.111 | 0.671 |
| **Model 3** | 0.238 | 0.337 | 0.136 | 0.601 | 0.073 | 0.773 |
| **Dependent variable** | **White Matter Lesion Loads** | | | | | |
| **Independent variable** | Beta value | P value | Beta value | P value | Beta value | P value |
| **Model 1** | -0.338 | 0.091 | -0.315 | 0.106 | -0.389 | 0.043 |
| **Model 2** | -0.309 | 0.153 | -0.284 | 0.186 | -0.377 | 0.074 |
| **Model 3** | -0.304 | 0.145 | -0.277 | 0.181 | -0.369 | 0.075 |

Model 1: controlled for Alzheimer's Disease Assessment Scale-Cognitive Subscale and age, education, gender, total intracranial volume;

Model 2: controlled for clinical dementia rating scale sum of boxes and age, education, gender, total intracranial volume;

Model 3: controlled for Composite memory score and age, education, gender, total intracranial volume;

Beta value=standardized coefficients, *p<0.05
